# Feasibility, reliability and validity of self-measurement of knee range-of-motion using an accelerometer-based smartphone application by patients with total knee arthroplasty

**DOI:** 10.1371/journal.pone.0307219

**Published:** 2024-10-03

**Authors:** Eleanor Shuxian Chew, Ee-Lin Woon, Juanita Krysten Miao-Shi Low, Luke Jonathan Haseler, Ismahfaris Ismail, Muhammad Bukhari Alif, Yu-Heng Kwan, John Wei-Ming Tan, Samantha Shi-Man Koh, Hee-Nee Pang, Jerry Delphi Yongqiang Chen, Seng-Jin Yeo, Shi-Ying Lim, Celia Ia-Choo Tan, Yong-Hao Pua

**Affiliations:** 1 Department of Physiotherapy, Singapore General Hospital, Singapore, Singapore; 2 School of Allied Health Care, Curtin University, Perth, Australia; 3 School of Computing, National University of Singapore, Singapore, Singapore; 4 Programme in Health Services and System Research, Duke-NUS Medical School, Singapore, Singapore; 5 Department of Orthopedic Surgery, Singapore General Hospital, Singapore, Singapore; 6 Medicine Academic Programme, Duke-NUS Graduate Medical School, Singapore, Singapore; Università degli Studi di Milano: Universita degli Studi di Milano, ITALY

## Abstract

**Aims:**

Limited knee range-of-motion (ROM) is common following total knee arthroplasty (TKA). It is associated with functional limitations and patient dissatisfaction. Regular knee ROM assessment is important but accurate testing traditionally requires timely access to trained healthcare professionals. Although accelerometer-based smartphone goniometry has shown to provide reliable and valid joint angles, current evidence of its use still positions healthcare providers as end users instead of patients themselves. Therefore, to maximize the impact of smartphone goniometry on post-TKA care, our study aimed to examine the feasibility, reliability, and validity of patients’ self-measurement of knee ROM using an accelerometer-based smartphone goniometry application.

**Methods:**

Patients were given standard instructions with a practice trial before the actual measurements. Passive knee flexion and extension ROM was measured on 2 sessions in 30 patients with TKA using 4 block-randomized methods: (i) smartphone self-assessment, (ii) long-arm goniometry by physiotherapist, (iii) smartphone assessment by physiotherapist, and (iv) extendable-arm goniometry by physiotherapist with placement adjudication. Feasibility was assessed by the number of participants who could independently perform the self-measurement. To assess intra- and inter-session reliability, we computed intraclass correlation coefficients (ICCs) from random-effects models. To assess intra- and inter-session agreement, we computed mean absolute differences (MADs) and minimum detectable change (MDC). To assess concurrent validity, we designated extendable-arm goniometry as the "gold standard" and compared other methods against it using ICCs and MADs.

**Results:**

All patients were able to comprehend and execute the assessment. 87% (n = 26) found the application easy to administer. Smartphone goniometry by patients showed excellent intra- and inter-session reliability (ICCs>0.97) and minimum variability (MAD = 0.9°-3.9°; MDC_95_ = 3.1°–9.0°). Smartphone or long-arm goniometry by physiotherapists did not outperform patients’ self-assessment (ICC = 0.96–0.99, MAD = 0.7°-3.1°; MDC_95_ = 2.2°-8.0°). Compared against extendable-arm goniometry, smartphone goniometry by patients measured knee flexion and extension ROM with a MAD of 4.5° (ICC, 0.97) and 2.2° (ICC = 0.98), respectively.

**Conclusion:**

Our study demonstrates that smartphone goniometry is feasible, reliable and accurate, and can be used with confidence in the self-assessment of knee ROM post-TKA. Future studies should further explore its utility in telemonitored rehabilitation, and its possible integration into mobile health applications to enhance accessibility to care following TKA.

## Introduction

Total knee arthroplasty (TKA) is a cost-effective surgical intervention for painful knee osteoarthritis in adults aged 65 years and over [1, 2]. However, limited knee range-of-motion (ROM) is common following a TKA. This is associated with functional limitations [3–5] and patient dissatisfaction [4, 5]. Accurate and regular knee ROM assessment is therefore important to inform recovery progress, identify at-risk patients [6], and guide rehabilitation decisions post-TKA.

Knee ROM is traditionally measured by trained professionals using the universal long-arm goniometer. Although the long-arm goniometer has demonstrated reasonable levels of reliability and validity in numerous studies to quantify knee ROM [7, 8], it requires a trained assessor to perform the measurement. This is manpower exhaustive. Furthermore, the demand for TKAs has been rapidly rising globally [9–11], and this is projected to increase by 673% from 2005 to 2030 in the United States [12]. This translates to an increased need for timely post-operative and rehabilitative care, further straining health systems. As such, there is an urgent need for a cost-effective and accurate assessment of knee ROM to reduce the dependence on healthcare resources without compromising on post-operative care and recovery following TKA.

Currently, developments in smartphone technology and software have provided smartphones the ability to measure joint ROM reliably [13]. Reliable and valid joint angles can be derived from the smartphone’s in-built accelerometer and trigonometric function [8, 13–17]. This may circumvent the difficulties of manually identifying anatomical landmarks for knee ROM measurement with a long-arm goniometer, thus removing the need for a trained assessor. Despite these proposed benefits of smartphone goniometry for self-assessment of joint ROM, current evidence of its use still positions healthcare providers as end users [8, 13–17]. No studies have evaluated the feasibility, reliability and validity as an assessor-free method, nor in patients more than 2 weeks post-TKA [13] where regular measurements are required [18].

Therefore, to maximize the impact of smartphone goniometry on post-TKA care, our study aimed to examine the feasibility, reliability and validity of patients’ self-measured knee ROM post-TKA using an accelerometer-based smartphone goniometry application.

## Methods

### Participants

A sample of 30 individuals aged 45 years or older who have had primary unilateral TKA in Singapore General Hospital within the last 16 weeks were consecutively recruited during their outpatient physiotherapy visit. Patients included in this study had to be able to provide informed consent and demonstrate the ability to hold and tap on the screen of a smartphone. Patients were excluded if they had cognitive dysfunction, communicable disease(s), or post-TKA complications. This study was approved by the SingHealth Centralised Institutional Review Board (CIRB) (CIRB Ref No: 2021/2479, Singapore) and patients provided written informed consent.

### Instrumentation

#### Smartphone application

**Fig 1:**
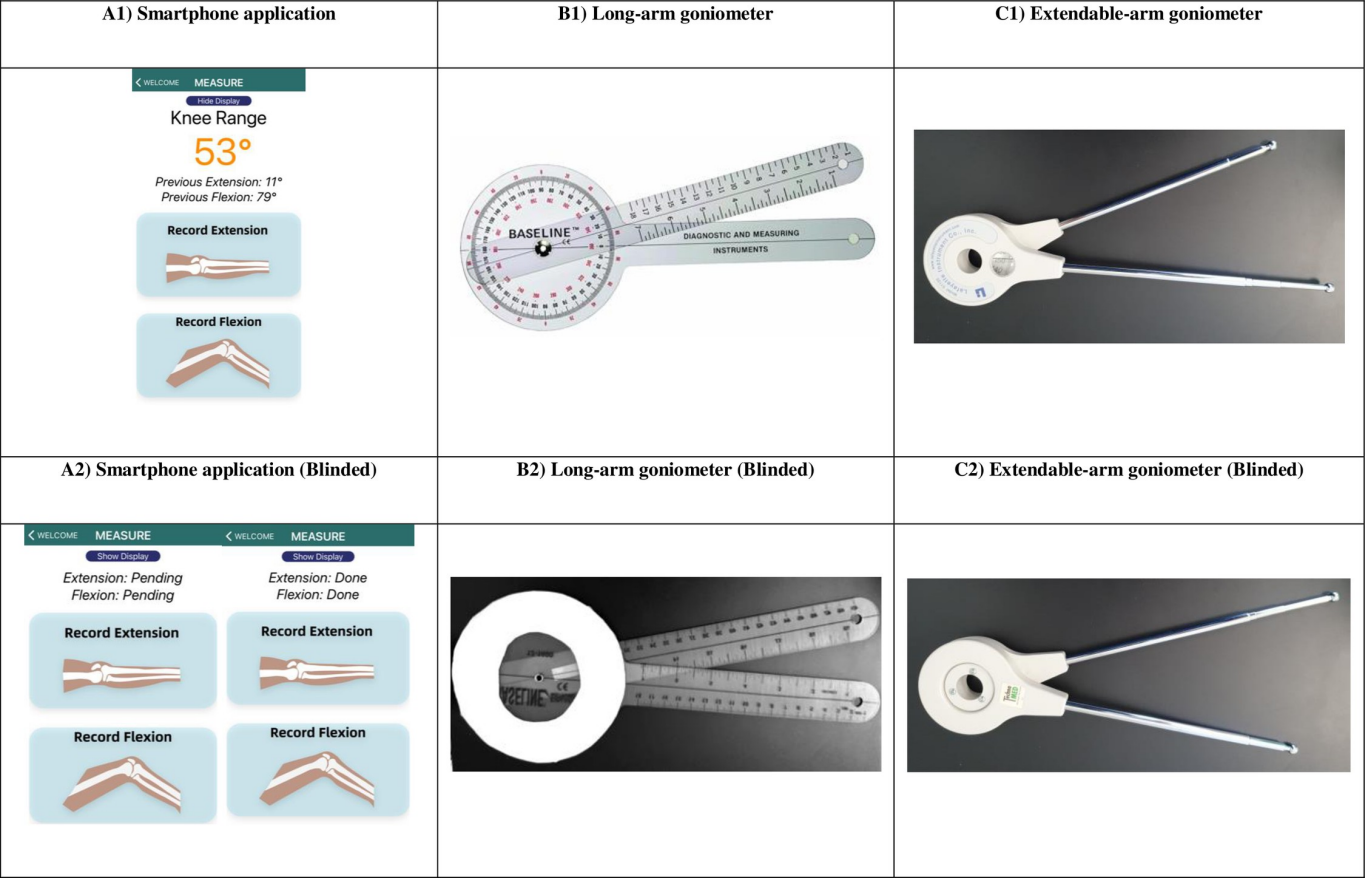
Standard instruments were used to compare the measurement of knee ROM. Efforts to ensure blinding of the primary assessor to the ROM angle include (A) feature on the smartphone application to hide and unhide, (B) opaque sheet covering one side of the long-arm goniometer and (C) flipping of the extendable-arm goniometer.

The smartphone self-assessment utilises an accelerometer-based application that was developed in-house based on trigonometric functions that converts shank angle to knee joint angle. This technique, first described by Ockendon & Gilbert [15] can be created and applied to any smartphone device containing accelerometers. The application was installed on a Samsung Galaxy S10 android smartphone (Samsung; Seoul, South Korea). We included older-adults friendly features on the user interface such as larger buttons and fonts, text labelled icons to ensure ease of self-measurement for the user (Fig 1A1).

#### Long-arm goniometer

We used a single plastic Baseline 12–1000 goniometer (Baseline Evaluation Instruments; New York, USA) with an 18inch movable arm and a 360° face for this study (Fig 1B1).

#### Extendable-arm goniometer

For our reference method, a Lafayette Gollehon extendable-arm goniometer model 01135 (Lafayette Instrument; Indiana, USA) with chromium-plated brass arms of 20-70cm that allowed for 360° arm rotation was used (Fig 1C1).

### Procedure

Four methods were evaluated: (i) smartphone self-assessment, (ii) long-arm goniometry by physiotherapist, (iii) smartphone assessment by physiotherapist, and (iv) extendable-arm goniometry by physiotherapist with placement adjudication (reference criterion).

For all patients, passive knee flexion ROM was measured before passive knee extension ROM (knee “position”). For knee flexion ROM, in long-seating, patients were asked to bring their leg to maximal knee flexion with their arms. For knee extension ROM, in long-seating, a patient’s leg was propped with a towel roll placed under the heel to ensure the soft tissues of the thigh and calf were clear off the plinth and the knee was fully extended. To ensure that patient’s hamstring would not limit patient’s ability to reach forward for self-measurement, the opposite leg was allowed to bent off the side of the plinth.

For each knee ROM position (flexion or extension ROM), method (i) was always done first to assess the patient’s ability to perform the test independently without reminder. Methods (ii) and (iii) were block randomized to remove any potential order effects. Method (iv) was done last. For each method and knee ROM position, 2 measurements were obtained. Following the collection of one round ("session") of flexion and extension ROM assessment, each patient performed another round ("session") of ROM assessment. Pain levels were monitored throughout the entire session to ensure they remain fairly constant.

Two physiotherapists who are familiar with the management of TKA were involved in the data collection as assessors: the primary assessor performed measurements for all patients and the secondary assessor performed data scribing. To minimize bias, efforts to ensure that assessors were blinded for each method are described in subsequent sections.

#### Method (i)—Smartphone self-assessment

**Fig 2:**
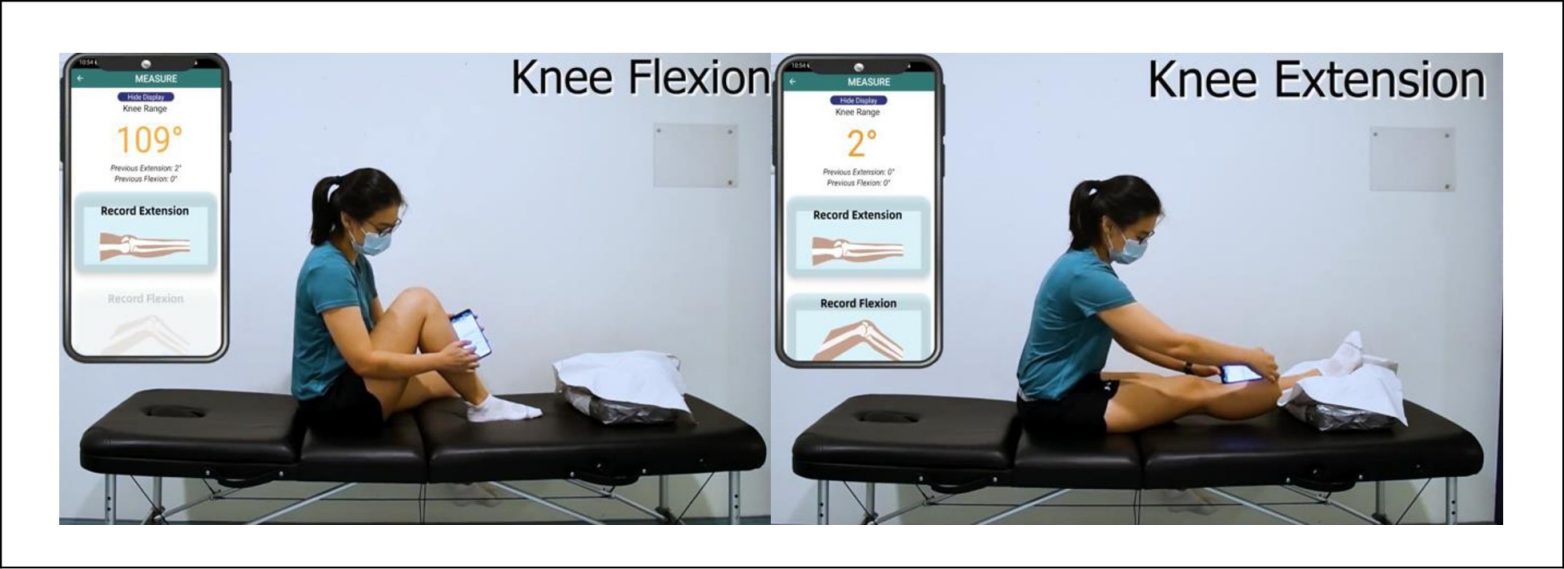
Smartphone goniometry self-assessment.

Patients were given standard instructions on how to perform the self-assessment using the smartphone application and then asked to repeat once under the supervision and guidance of a physiotherapist before the actual measurement. In maximal knee flexion and extension, the edge of the smartphone was placed against the patient’s skin parallel to and over the middle aspect of the tibia before tapping on the button on the screen to capture the angle (Fig 2). To ensure blinding of the patient, the smartphone application was modified for this study so that the angles do not appear during the ROM measurement (Fig 1A2). The secondary assessor would reveal the recorded measurement for data collection by selecting the ‘unhide’ option.

Feasibility was assessed in two ways: (a) by the number of patients who could independently perform the self-measurement and (b) by the patients’ self-ratings of perceived difficulty with independent home use on a simple 11-point Likert scale. A score of 0 means no “no difficulties”, and 10 means “extremely difficult”. Patients were invited to provide additional comments if they found the self-assessment difficult.

#### Method (ii)—Long-arm goniometry by physiotherapist

The axis of the goniometer was centred on the lateral femoral epicondyle, with the proximal arm aligned with the greater trochanter and the distal arm aligned with the lateral malleolus [19]. To ensure blinding of the primary assessor, the side of the goniometer facing the assessor was covered with an opaque sheet (Fig 1B2). The secondary assessor recorded the reading taken from the other side of the goniometer.

#### Method (iii)—Smartphone assessment by physiotherapist

The edge of the smartphone was placed against the patient’s skin, parallel to and over the middle aspect of the tibia by the physiotherapist, before the button on the screen was tapped to capture the knee ROM. The same blinding process described in method (i) was performed to obtain knee ROM measures (Fig 1A2).

#### Method (iv)—Extendable-arm goniometry by physiotherapist with placement adjudication

Knee ROM was assessed using an extendable-arm goniometer with 2 trained assessors agreeing on the placement as ‘gold standard’. Landmarks for the long-arm and extendable-arm goniometer were as follows: fixed arm pointing to or over the greater trochanter of the femur axis at the lateral epicondyle of the femur and moving arm pointing toward or over the lateral malleolus of the fibula [19]. To ensure blinding of the 2 assessors, the extendable-arm goniometer was flipped such that the side of the goniometer with the measurement faced away during the assessment (Fig 1C2). After each measurement, the goniometer was passed to the secondary assessor who logged the measurement.

### Sample size calculation

Using the ‘ICC.Sample.Size’ R package [20, 21], a minimum sample size of 26 participants was calculated to be required to test whether an anticipated reliability of 0.90 [7, 22–24] for inter-session goniometry exceed a minimum acceptable reliability of 0.75, at 0.05 alpha level (1-tailed) and 80% power. To ensure adequate power, we recruited 30 participants.

### Statistical analysis

#### Feasibility

To assess the feasibility of smartphone goniometry, we calculated descriptive statistics for the number of patients who were unable to complete the test and their self-reported ratings.

#### Reliability and validity

Intraclass correlation (ICC) for intra-rater (intra-rater reliability), inter-session (test-retest reliability) and inter-method (validity) agreement was calculated from 2-way random-effects models. Specifically, for intra-rater agreement, ICC was based on a random-effects model for single measurement. For inter-session and inter-method agreement, ICC [2,2] was based on a mean-rating, random-effects model of absolute agreement. Intra-rater, inter-session and inter-method variability was measured in 2 ways. First, we computed the mean absolute differences (MADs) [25] between (i) any 2 measurements (measured for each patient) obtained within the same session (intra-rater variability) (ii) any 2 measurements obtained on different (test and retest) sessions (inter-session variability) and (iii) any 2 (session-averaged) measurements obtained from different methods (inter-method variability). For all MADs, 95%CIs were estimated using a bootstrapping procedure with 1000 iterations. Second, we used the root means square error (rMSE) from the fitted random-effects models to compute the minimum detectable change (MDC_95_) by multiplying rMSE and 2.77 (MDC_95_ = 2.77 x rMSE). Specifically, the MDC_95_ represents the magnitude of the maximum expected variability (difference) between repeated observations expected in 95% of pairs observations that are obtained (i) within each session (intra-rater variability), (ii) between sessions (inter-session variability), and (iii) between methods (inter-method variability).

All analyses were done in R (http://www.r-project.org) using the psych [26] and lme4 [27] packages.

## Results

A total of 30 TKA patients were studied, and their demographic and clinical characteristics are described in Table 1. All patients were able to ambulate without (human) assistance.

**Table 1:** Participant demographics and clinical characteristics.

Variables	All Participants (*n* = 30)
Age (years)	60.3 **66.0** 70.8 (65.3 ± 6.9)
Women	60% (18)
BMI (kg/m^2^)	24.1 **26.8** 31.5 (28.0 ± 5.5)
Premorbid walking aid used	
Quad stick	3% (1)
Walking stick	17% (5)
None	80% (24)
Weeks from surgery	6.0 **8.0** 12.8 (9.1 ± 5.8)
Knee ROM^a^	
Flexion (deg)	92.8 **101.9** 105.7 (92.8 ± 12.1)
Extension (deg)^b^	-1.1–**3.1–**5.1 (-4.5 ± 4.9)
Education Level	
Primary or lower	23% (7)
Secondary	40% (12)
Tertiary	37% (11)
Ethnic Group	
Chinese	73% (22)
Malay	13% (4)
Indian	13% (4)
Comorbidity	
Hypertension	63% (19)
Dyslipidemia	53% (16)
Diabetes	17% (5)

Continuous variables are summarized as 25^th^ **50**^th^ 75^th^ percentiles and mean ± SD.

Note that bold values are the median for continuous variables.

Categorical variables are summarized as percentages and counts (*n*).

^a^ Knee ROM was taken from referent method–extendable-arm goniometer.

^b^ Negative values indicated knee flexion contracture or lack of full knee extension.

### Feasibility

All patients were able to understand the instructions and self-administer the assessment independently. 87% (n = 26) indicated that it would be easy to use (8 or more) with none indicating that they would face difficulty to use (4 or less) smartphone goniometry at home.

### Reliability

Smartphone goniometry by patients showed excellent intra-rater and inter-session reliability (ICCs>0.97) and minimum variability (MAD = 0.9°-3.9°; MDC_95_ = 3.1°–9.0°) (Table 2).

**Table 2:** Intra-rater, inter-session, and inter-method agreement.

Method	Intra-rater agreement			Inter-session agreement			Inter-method agreement*		
	ICC (95% CI)	MAD, deg (95% CI)	MDC_95_, deg	ICC (95% CI)	MAD, deg (95% CI)	MDC_95_, deg	ICC (95% CI)	MAD, deg (95% CI)	MDC_95_, deg
Flexion									
Smartphone self-assessment	0.98 (0.96–0.99)	2.0 (1.4–2.6)	5.3	0.97 (0.94–0.99)	3.9 (2.9–4.9)	9.0	0.97 (0.94–0.98)	4.5 (3.8–5.2)	8.8
Smartphone	0.99 (0.98–0.99)	1.4 (1.1–1.9)	4.0	0.98 (0.95–0.99)	3.1 (2.3–4.1)	7.9	0.97 (0.94–0.98)	4.1 (3.3–5.0)	8.2
Long-arm goniometer	0.99 (0.98–0.99)	1.5 (1.2–1.7)	3.6	0.97 (0.95–0.99)	3.1 (3.2–4.1)	8.0	0.97 (0.94–0.98)	3.2 (2.4–4.3)	8.4
Extendable-arm goniometer	0.99 (0.98–1.00)	1.2 (0.9–1.5)	3.4	0.96 (0.91–0.98)	3.7 (2.7–4.9)	8.6			
Extension									
Smartphone self-assessment	0.97 (0.95–0.99)	0.9 (0.6–1.2)	3.1	0.98 (0.97–0.99)	1.3 (0.9–1.7)	3.3	0.98 (0.97–0.99)	2.2 (1.7–2.7)	3.0
Smartphone	0.96 (0.93–0.98)	1.1 (0.7–1.5)	3.2	0.98 (0.95–0.99)	1.4 (0.9–1.8)	3.5	0.98 (0.96–0.99)	1.6 (1.2–2.0)	3.1
Long-arm goniometer	0.96 (0.93–0.98)	0.7 (0.5–1.0)	2.2	0.97 (0.94–0.99)	1.0 (0.6–1.3)	2.4	0.98 (0.96–0.99)	1.4 (1.0–1.8)	2.6
Extendable-arm goniometer	0.97 (0.95–0.99)	0.8 (0.7–1.0)	2.2	0.98 (0.96–0.99)	1.3 (0.8–1.5)	2.7			

Abbreviations: ICC, intraclass correlation; CI, confidence interval; MDC_95_, minimum detectable change at 95% confidence level; MAD, mean absolute difference.

* Extendable-arm goniometer taken as the referent.

### Validity

Physiotherapist assessment using smartphone or long-arm goniometry (inter-method agreement), did not outperform patient self-assessment (ICC = 0.96–0.99, MAD = 0.7°-3.1°; MDC_95_ = 2.2°-8.0°) (Table 2). Compared against extendable-arm goniometry, smartphone goniometry by patients measured knee flexion and extension ROM with a MAD of 4.5° (ICC, 0.97) and 2.2° (ICC = 0.98), respectively.

## Discussion

Self-assessment of knee ROM using smartphone goniometry was feasible in patients post-TKA. Our study demonstrated excellent intra-rater reliability, inter-session reliability with acceptable validity when compared to extendable-arm goniometry with placement adjudication as ‘gold standard’. To our knowledge, this study represents the first effort to explore the utility of smartphone goniometry for patient self-assessment of knee ROM after TKA.

### Feasibility

All patients were able to comprehend and execute the smartphone goniometry measurement according to the instructions provided.

### Reliability

Our findings were similar to previous literature [8, 28] for intra-rater reliability (ICC = 0.97–0.98) of goniometer measurements for knee ROM of surgical orthopaedic patients. In our study, self-assessment using smartphone goniometry showed similar intra-rater reliability to measurements obtained by physiotherapists using smartphone goniometry, long-arm goniometer or an extendable-arm goniometer.

The smartphone application in our study utilised the most common method, first described by Ockendon & Gilbert [15]. Previously known as Knee Goniometer, studies that applied similar methods of smartphone goniometry as our study reported superior and excellent intra-rater reliability in healthy adults (ICC = 0.90–0.99) [14–16]. Further exploring the intra-rater reliability of smartphone goniometry compared to standard goniometry in a clinical population, Pereira et al. [14] evaluated the application on patients immediately after unicompartmental knee arthroplasty and TKA. Similar to our study, intra-rater reliability was found to be good-to-excellent for passive knee flexion (ICC = 0.87) and extension (ICC = 0.87) ROM, despite reported limitations from compressive bandages that could potentially limit accurate measurement of postoperative knee ROM with either testing instrument [14]. However, authors in this study had only explored intra-rater reliability of application use within clinicians. Our study thus adds to the evidence that patients’ self-measurement of knee ROM post-TKA is possible with an accelerometer-based smartphone application.

### Validity

Our findings for assessment done using a smartphone accelerometer-based application evaluating knee ROM by clinicians and therapists were consistent with previous studies [13–17]. As expected, MAD and MDC_95_ were highest for self-measurement using smartphone goniometry, but the difference is unlikely to be clinically important, and therefore still adequately valid. Therefore, our findings extend previous study results to measurements performed by laypeople and to patients beyond the first 2 weeks after TKA surgery.

### Limitations

Our study has limitations. Getting a gold standard is always a challenge. While radiographs are used as the reference standard in some studies, exposure of subjects to radiation for the purpose of assessing goniometry devices would be excessive. There are also issues associated with interpreting results measuring joint angles from a radiograph due to the angle of the camera relative to the subject [29]. All the previous studies done to evaluate knee smartphone goniometry have therefore used long-arm goniometers as the reference standard for validation of different goniometers [13]. We have therefore devised an alternative referenced measure and would be the first to compare smartphone goniometry against extendable-arm goniometry with placement adjudication by a second assessor as gold standard. We have also included blinding to ensure rigour in our analysis to ensure confidence in our criterion instrument and our validation study. Second, extreme ROM measures of knee flexion (maximum of 145°) and extension (maximum of 0°) [15] was not possible on a smartphone. However, as patients in our study were examined in the early stages of recovery post-TKA, it was unlikely that knee ROM would breach the defined measurement limits. Third, results cannot be generalized to smartphone goniometry of the knee in non-clinical environments as only one smartphone model was used in our study. As previous studies have shown that accelerometer-based goniometry across different makes of smartphones were unlikely to affect testing accuracy [13–16], the limitation in testing environment can be overcome with the provision of clear instructions to replicate the assessment procedure. Lastly, inter-session reliability was obtained with measurement sessions performed on the same visit, differing from conventional test-retest reliability methods. As our study population was expected to present with different knee ROM measurements on separate visits [18], it was not feasible for the sessional ROM measurements to be obtained this way, especially when the study’s focus was on the patients’ ability to repeat the self-measurement.

### Clinical implication

Our study carries significant implications for clinical practice. By demonstrating the interchangeability of smartphone goniometry between clinicians and patients, our findings suggest that patients can more frequently self-measure their knee ROM post-TKA. This capability not only empowers patients to actively engage in their recovery process but also provides clinicians with valuable data to monitor progress remotely [30].

Beyond providing feedback to patients about their post-surgical progress, regular ROM assessment potentially fosters motivation [31] during rehabilitation. Moreover, with the integration of tele-rehabilitation or video consultations facilitated by technological advancements, remote monitoring becomes feasible. Accurate measurement of knee ROM becomes paramount in supporting such innovative models of care, as changes in ROM can signify complications such as stiffness, joint contractures, or implant malposition. Early detection of these issues enables timely interventions [30], thereby optimizing clinical outcomes.

## Conclusion

In conclusion, our study demonstrates that smartphone goniometry is feasible, reliable and accurate, and can be used with confidence in the self-assessment of knee ROM post-TKA. Future studies should further explore its utility in telemonitored rehabilitation, and its possible integration into existing mobile health applications to enhance accessibility to care following TKA.
